# Waist Gain Is Associated with a Higher Incidence of Nonalcoholic Fatty Liver Disease in Korean Adults: A Cohort Study

**DOI:** 10.1371/journal.pone.0158710

**Published:** 2016-07-15

**Authors:** Kyung Eun Yun, Ga Eun Nam, Jisun Lim, Hye Soon Park, Yoosoo Chang, Hyun-Suk Jung, Chan-Won Kim, Byung-Joon Ko, Eun Chul Chung, Hocheol Shin, Seungho Ryu

**Affiliations:** 1 Center for Cohort Studies, Total Healthcare Center, Kangbuk Samsung Hospital, Sungkyunkwan University, School of Medicine, Seoul, South Korea; 2 Department of Family Medicine, Korea University Ansan Hospital, Korea University College of Medicine, Ansan-si, South Korea; 3 Department of Family Medicine, Asan Medical Center, University of Ulsan College of Medicine, Seoul, South Korea; 4 Department of Radiology, Kangbuk Samsung Hospital, Sungkyunkwan University, Seoul, South Korea; 5 Department of Family Medicine, Kangbuk Samsung Hospital, Sungkyunkwan University School of Medicine, Seoul, South Korea; 6 Department of Occupational and Environmental Medicine, Kangbuk Samsung Hospital, Sungkyunkwan University, School of Medicine, Seoul, South Korea; University of Verona, Ospedale Civile Maggiore, ITALY

## Abstract

**Background:**

We examined the relationship between changes in waist circumference (WC) and the incidence of nonalcoholic fatty liver disease (NAFLD).

**Methods:**

A cohort study of 37,130 men and women were followed-up annually or biennially. Differences in WC between baseline and subsequent measurements were categorized in quartiles: first (WC loss), second (no change in WC as the reference), third and highest quartiles (WC gain). The presence of fatty liver was determined using ultrasound. Parametric Cox modeling was used to estimate the adjusted hazard ratios (aHR) and 95% confidence intervals (CIs) of the incidence of NAFLD.

**Results:**

During 127,324.4 person-years of follow-up, 6249 participants developed NAFLD. Despite adjusting for possible confounders, the risk of development of NAFLD increased with increasing quartiles of WC change in a dose-response manner (p for trend < 0.001). Compared with the reference, WC loss was associated with a lower risk of NAFLD (men: aHR 0.79 [95% CI: 0.73–0.87]; women: 0.72 [0.63–0.81]), and the highest quartile (WC gain) was associated with a higher risk of NAFLD (men: 1.30 [1.19–1.42]; women: 1.48 [1.31–1.67]).

**Conclusion:**

Waist gain appears to increase the risk of developing NAFLD, independently of the baseline body mass index and WC.

## Introduction

Nonalcoholic fatty liver disease (NAFLD) has become one of the most common causes of chronic liver disease worldwide [1,2]. In addition to its potential to progress to cirrhosis or hepatocellular carcinoma [2], there is a body of evidence that NAFLD, as a precursor of the metabolic syndrome, is linked to a substantial increase in risk for metabolic complications such as diabetes and cardiovascular disease [3–7].

Abdominal obesity has been known as an important risk factor for NAFLD [8–10]. The increase in waist circumference (WC) is associated with visceral adipose tissue (VAT) accumulation, particularly individuals with a low body mass index (BMI) [11]. Large WC indicates abdominal adiposity, which is associated with a cluster of cardiometabolic risks [12] and is recognized as an important predictor of NAFLD, even among normal-weight individuals [13]. In a previous study, no other anthropometric parameters are independently related to NAFLD after adjusting for WC [14].

Although some cross-sectional studies have suggested that WC measured at one point in time is strongly associated with the risk of NAFLD [15–17], few cohort studies have prospectively examined the development of NAFLD [18]. Furthermore, the role of waist change over time in the development of NAFLD is largely unknown [15–18]. Thus, this prospective study aimed to determine if increases in WC over time contribute to a higher incidence of future NAFLD in Korean men and women.

## Materials and Methods

### Study population

The study population consisted of participants who underwent a comprehensive health screening that included an ultrasound liver examination between 2002 and 2013 at Kangbuk Samsung Hospital, Seoul, Korea. In Korea, the Industrial Safety and Health Law requires employees to participate in annual or biennial health examinations. 75,113 potential participants who completed WC measurement received ≥ 3 follow-up visits between 2002 and 2013.

**Fig 1:**
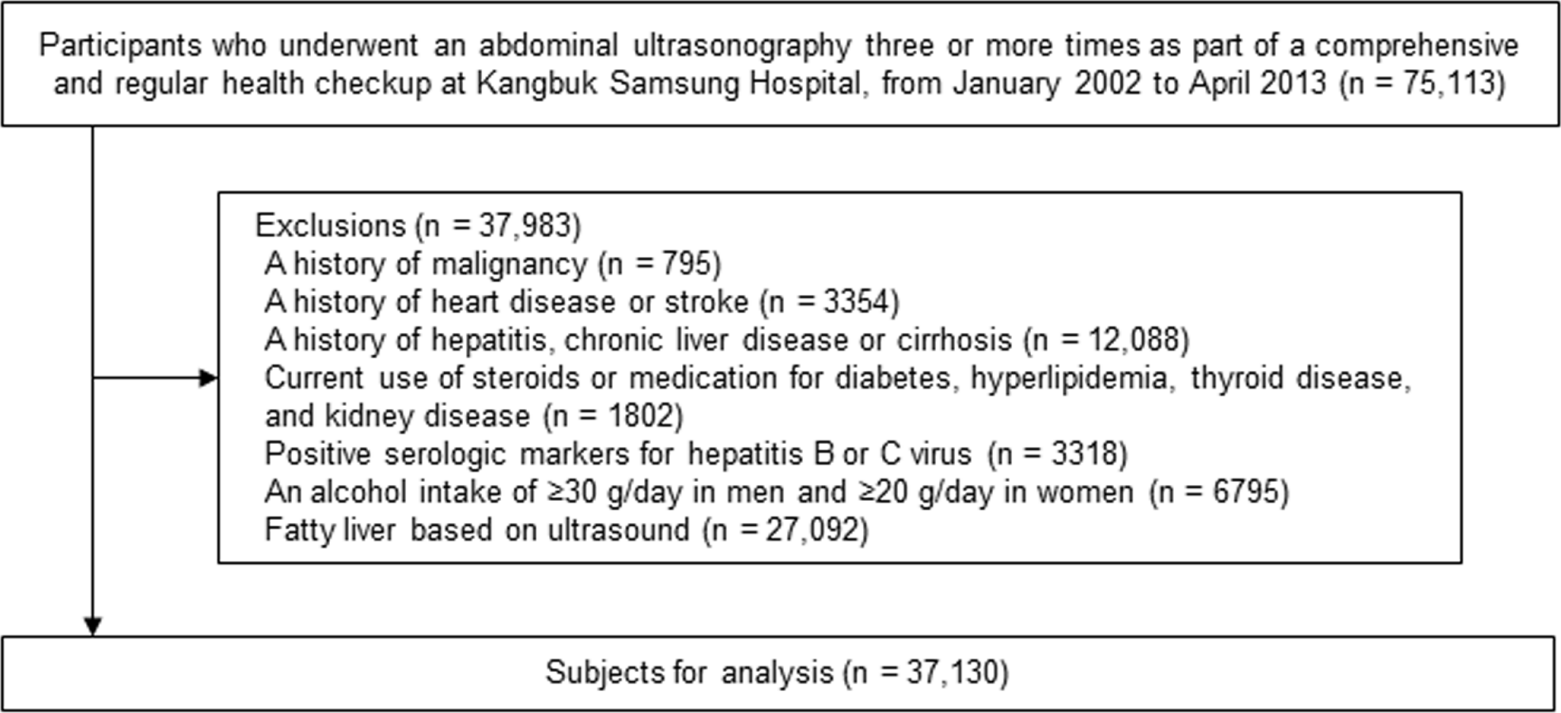
Flow diagram for identifying study participants. Because some individuals met ≥ 1 criterion for exclusion, 37,130 participants were eligible for inclusion.

We excluded participants with evidence of liver disease or other factors that could influence NAFLD traits or ultrasonographic liver findings (Fig 1), including: history of malignancy; history of cardiovascular disease; history of hepatitis, chronic liver disease, or cirrhosis; currently receiving steroids or medication for diabetes, hyperlipidemia, or thyroid disease; positive serological markers for hepatitis B or C virus; alcohol intake ≥ 30 g/day in men or ≥ 20 g/day in women; and ultrasonographically detected fatty liver at visit 1 or 2. Because some individuals met ≥ 1 criterion for exclusion, 37,130 participants were eligible for inclusion. This study was approved by the institutional review board of Kangbuk Samsung Hospital, which waived the requirement for informed consent because we only accessed data that had been de-identified.

### Measurements

Data on medical history, medication use, and health-related behaviors were collected using a self-administered questionnaire. Physical characteristics and serum biochemical parameters were measured as previously described [19,20]; all data were collected during health examinations. Details regarding alcohol use, including the frequency of intake per week and the average amount of intake per episode, were also collected. Current smokers were identified, and the weekly frequency of physical activity was assessed. Body weight was measured in light clothing and no shoes to the nearest 0.1 kg using a digital scale. Height was measured to the nearest 0.1 cm. Body mass index (BMI) was calculated as weight in kilograms divided by height in meters squared (kg/m^2^). WC was measured at the midpoint between the bottom of the rib cage and the top of the iliac crest to the nearest 0.1 cm as participants were standing with their weight equally distributed on both feet, with their arms at their sides and their head facing forward. Trained nurses measured sitting blood pressure using standard mercury sphygmomanometers.

Blood samples were taken from the antecubital vein after at least a 10-h fast. Serum glucose, lipid profiles and liver enzymes were measured using Bayer reagent packs and an automated chemistry analyzer (Advia 1650TM Autoanalyzer; Bayer Diagnostics, Leverkusen, Germany) as previously described [19,20]. Measurements were taken using the hexokinase method for glucose and the enzymatic colorimetric assay for serum lipids. Insulin and high-sensitivity C-reactive protein (hsCRP) were measured using an immunoradiometric assay (Biosource, Nivelles, Belgium) and using particle-enhanced immunonephelometry and the BNIITM System (Dade Behring, Marburg, Germany), respectively. Insulin resistance was assessed using the homeostasis model assessment of insulin resistance (HOMA-IR): HOMA-IR = fasting blood insulin (μU/mL) × fasting blood glucose (mmol/L) / 22.5. The Laboratory Medicine Department at Kangbuk Samsung Hospital, Seoul, Korea is accredited by the Korean Society of Laboratory Medicine (KSLM) and the Korean Association of Quality Assurance for Clinical Laboratories (KAQACL). This laboratory participates in the proficiency testing surveys conducted by the College of American Pathologists.

Abdominal ultrasounds were performed using a Logic Q700 MR 3.5-MHz transducer (GE, Milwaukee, WI, USA) by experienced radiologists, all of whom were unaware of the study aims. Images were captured in a standard manner (i.e., the patient was in the supine position with the right arm raised above the head). An ultrasonographic diagnosis of fatty liver was made if a diffuse increase in fine echoes in the liver parenchyma was noted in comparison with the kidney or spleen parenchyma [21]. The interobserver reliability and intraobserver reliability for fatty liver diagnosis were substantial (κ statistic = 0.74) and excellent (κ statistic = 0.94), respectively [22]. NAFLD was defined as the presence of fatty liver in the absence of excessive alcohol use (a threshold of < 30 g/d was used for men and < 20 g/d for women) [23] or other identifiable causes, as described in the exclusion criteria.

### Statistical analyses

Descriptive statistics were used to summarize participant characteristics according to the quartile for WC changes in men and women. We evaluated distributions of all continuous variables and the appropriate transformations were performed as needed during analysis.

The change in WC was calculated for each participant as the difference in WC between visit 2 and baseline (visit 1). Change in WC was classified into the following quartiles: ≤ -2.0 cm (WC loss), -1.9–0.5 cm (no change in WC as the reference category), ≥ 0.6–3.2 cm and ≥ 3.3 cm (third and highest quartiles as WC gain) for men; or ≤ -1.5 cm (WC loss), -1.4–1.3 cm (no change in WC as the reference category), 1.4–4.3 cm and ≥ 4.4 cm (third and highest quartiles as WC gain) for women. The baseline data according to quartiles of changes in WC were compared by using one-way analysis of variance for continuous variables or chi-square test for categorical variables. Person-years were calculated as the sum of the follow-up duration from visit 2 until the assumed time of fatty liver development or each individual’s final examination. Since we knew that NAFLD had developed between the two visits, but did not know the precise timing, we used parametric Cox modeling to take interval censoring into account (stpm command in STATA) [24]. Using these models, the baseline hazard function was parameterized with restricted cubic splines in log time with 4 degrees of freedom. We estimated the adjusted hazard ratios (aHR) with 95% confidence intervals (CI) for incidental NAFLD according to WC changes.

We first estimated hazard ratios with a 95% CI to determine the association between the baseline WC (measured at visit 1) and the risk of developing NAFLD, adjusted for baseline potential covariates (e.g., age, smoking, alcohol intake, exercise, educational level, and BMI). Then, we adjusted for other metabolic markers (e.g., triglycerides, high-density lipoprotein cholesterol [HDL-C], glucose, systolic blood pressure, HOMA-IR, and hsCRP levels). To analyze the association between WC change between visits 1 and 2 and the risk of developing NAFLD, the models were initially adjusted for age, period of WC change, smoking, alcohol intake, exercise, educational level, BMI, and then baseline WC. To determine the linear risk trends, the number of quartiles was used as the continuous variable and was tested for each model. We assessed the proportional hazards assumption by examining graphs of estimated log(-log)(*SURVIVAL*).

Statistical analysis was performed using STATA version 11.2 (StataCorp LP, College Station, TX). All reported p values are two-tailed, and p < 0.05 was considered statistically significant.

## Results

19,921 women (53.7%) and 17,209 men (46.3%) were enrolled in this study. The means (standard deviation) of age and WC were 39.4 years (7.3) and 80.3 cm (6.4) for men, and 38.6 years (7.0) and 71.1 cm (6.5) in women, respectively. The baseline characteristics of the study participants in relation to the changes in the WC categories are outlined in Tables 1 and 2. For both men and women, age, baseline WC, BMI, systolic and diastolic blood pressure, total cholesterol, triglycerides, alanine aminotransferase (ALT), gamma glutamyltransferase (GGT), hsCRP, and HOMA-IR were inversely associated with WC change quartiles. For men, the proportion of current smokers was positively associated with WC change quartiles. For women, low-density lipoprotein cholesterol (LDL-C) and the proportion of nondrinkers were inversely associated with WC change quartiles.

**Table 1:** Baseline characteristics of male study participants based on changes in waist circumference (cm) during the 2-year period between visit 1 and visit 2.

Variables	Overall	Quartiles for WC change (cm) during the 2 years from visit 1 to visit 2				
		1^st^ Q	2^nd^ Q	3^rd^ Q	4^th^ Q	P value
		(≤ -2.0 cm)	(-1.9–0.5 cm)	(0.6–3.2 cm)	(≥ 3.3 cm)	
No.	17,209	4627	3998	4286	4298	
Age (y)^a^	39.4 (7.3)	39.8 (7.5)	39.7 (7.2)	39.5 (7.3)	38.5 (7.2)	<0.001
WC (cm) at visit 1^a^	80.3 (6.4)	83.0 (6.1)	80.7 (6.1)	79.4 (6.2)	77.9 (5.9)	<0.001
WC (cm) at visit 2^a^	80.8 (6.4)	78.4 (6.0)	80.1 (6.1)	81.3 (6.2)	83.7 (6.0)	<0.001
BMI (kg/m^2^)^a^	22.9 (2.3)	23.3 (2.3)	23.0 (2.3)	22.8 (2.3)	22.7 (2.2)	0.009
Systolic BP (mmHg)^a^	114.6 (12.6)	115.0 (13.0)	114.7 (12.7)	114.5 (12.4)	114.3 (12.4)	<0.001
Diastolic BP (mmHg)^a^	75.5 (8.9)	75.9 (9.1)	75.6 (8.8)	75.3 (8.8)	75.3 (8.9)	<0.001
Fasting glucose (mg/dL)^a^	91.7 (11.2)	92.2 (11.6)	92.0 (10.7)	91.5 (10.7)	91.3 (11.2)	<0.001
Total cholesterol (mg/dL)^a^	192.0 (31.6)	194.8 (32.1)	193.0 (31.8)	190.9 (31.1)	189.0 (31.0)	<0.001
LDL-C (mg/dL)^a^	114.1 (27.3)	115.5 (27.5)	115.1 (27.9)	113.5 (27.0)	112.2 (26.7)	0.088
HDL-C (mg/dL)^a^	54.6 (11.1)	54.4 (11.0)	54.7 (11.1)	54.5 (11.1)	54.9 (11.2)	<0.001
Triglycerides (mg/dL)^b^	102 (77–140)	108 (80–145)	102 (77–140)	100 (76–137)	98 (74–134)	<0.001
ALT (U/I)^b^	21 (17–27)	22 (18–28)	21 (17–28)	21 (17–26)	20 (16–26)	<0.001
AST (U/I)^b^	22 (19–26)	23 (20–27)	22 (19–26)	22 (19–25)	21 (19–25)	<0.001
GGT (U/I)^b^	22 (16–33)	23 (16–35)	22 (16–33)	22 (16–33)	21 (16–31)	<0.001
hsCRP (mg/L)^b^	0.4 (0.1–0.8)	0.4 (0.1–0.8)	0.4 (0.1–0.8)	0.4 (0.1–0.8)	0.4 (0.1–0.8)	0.001
HOMA-IR^b^	1.6 (1.3–2.1)	1.7 (1.3–2.1)	1.7 (1.3–2.1)	1.6 (1.2–2.0)	1.6 (12–2.0)	<0.001
Current smoker (%)	37.1	34.7	36.4	37.1	40.3	<0.001
Nondrinker (%)	16.2	16.3	16.2	16.4	15.9	0.701
Regular exerciser (%)^c^	18.2	17.9	18.7	17.7	18.4	0.790

Abbreviations: WC, waist circumference; BMI, body mass index; BP, blood pressure; LDL-C, low-density lipoprotein-cholesterol; HDL-C, high-density lipoprotein-cholesterol; ALT, alanine aminotransferase; AST, aspartate aminotransferase; GGT, γ-glutamyltranspeptidase; hsCRP, high-sensitivity C-reactive protein; HOMA-IR, homeostasis model assessment of insulin resistance.

^a^Data shown as the mean (standard deviation).

^b^Data shown as the median (interquartile range).

^c^Considered ≥ 3 times/week.

**Table 2:** Baseline characteristics of female study participants based on changes in waist circumference (cm) during the 2-year period between visit 1 and visit 2.

Variables	Overall	Quartiles for WC change (cm) during the 2 years between visit 1 and visit 2				
		Q1	Q2	Q3	Q4	P value
		(≤ -1.5 cm)	(-1.4–1.3 cm)	(1.4–4.3 cm)	(≥ 4.4 cm)	
No.	19,921	5115	4857	4975	4974	
Age (y)^a^	38.6 (7.0)	39.0 (7.3)	38.8 (7.0)	38.7 (6.9)	38.1 (6.9)	<0.001
WC (cm) at visit 1^a^	71.1 (6.5)	74.4 (6.6)	71.1 (6.1)	69.9 (6.0)	69.0 (5.9)	<0.001
WC (cm) at visit 2^a^	72.5 (6.7)	69.8 (6.2)	71.1 (6.1)	72.7 (6.1)	76.5 (6.4)	<0.001
BMI (kg/m^2^)^a^	21.3 (2.3)	21.7 (2.4)	21.2 (2.3)	21.1 (2.3)	21.1 (2.3)	0.009
Systolic BP (mmHg)^a^	107.9 (12.7)	108.8 (13.2)	107.9 (12.6)	107.9 (12.6)	107.1 (12.4)	<0.001
Diastolic BP (mmHg)^a^	69.3 (8.8)	69.8 (9.0)	69.2 (8.8)	69.4 (8.7)	68.8 (8.6)	<0.001
Fasting glucose (mg/dL)^a^	89.1 (8.5)	89.4 (8.4)	89.0 (8.5)	89.0 (8.4)	89.2 (8.6)	0.168
Total cholesterol (mg/dL)^a^	183.6 (30.7)	185.5 (31.5)	183.8 (30.3)	183.4 (30.6)	181.5 (30.3)	<0.001
LDL-C (mg/dL)^a^	102.5 (26.1)	104.7 (26.6)	102.7 (25.7)	102.0 (26.3)	100.6 (25.7)	<0.001
HDL-C (mg/dL)^a^	62.0 (12.8)	61.9 (12.9)	62.0 (13.0)	62.4 (12.8)	61.8 (12.5)	0.801
Triglycerides (mg/dL)^b^	73 (57–96)	75 (58–100)	73 (58–97)	72 (56–96)	72 (57–94)	<0.001
ALT (U/I)^b^	15 (12–19)	15 (12–19)	15 (12–19)	15 (12–18)	15 (12–18)	0.003
AST (U/I)^b^	20 (17–23)	20 (17–23)	19 (17–23)	20 (17–23)	20 (17–23)	0.686
GGT (U/I)^b^	11 (9–15)	12 (9–15)	11 (9–15)	11 (8–15)	11 (8–15)	<0.001
hsCRP (mg/L)^b^	0.2 (0.1–0.5)	0.3(0.1–0.6	0.2 (0.1–0.5)	0.2 (0.1–0.5)	0.2 (0.1–0.5)	<0.001
HOMA-IR^b^	1.7 (1.4–2.2)	1.8 (1.4–2.2)	1.7 (1.4–2.2)	1.7 (1.4–2.2)	1.7 (1.4–2.2)	0.004
Current smoker (%)	2.4	2.3	2.4	2.4	2.7	0.243
Nondrinker (%)	67.1	68.1	66.9	67.3	65.9	0.043
Regular exerciser (%)^c^	17.7	17.6	18.1	17.7	17.3	0.598

Abbreviations: WC, waist circumference; BMI, body mass index; BP, blood pressure; LDL-C, low-density lipoprotein-cholesterol; HDL-C, high-density lipoprotein-cholesterol; ALT, alanine aminotransferase; AST, aspartate aminotransferase; GGT, γ-glutamyltranspeptidase; hsCRP, high-sensitivity C-reactive protein; HOMA-IR, homeostasis model assessment of insulin resistance.

^a^Data are shown as the mean (standard deviation).

^b^Data are shown as the median (interquartile range)

^c^Considered ≥ 3 times/week.

Table 3 indicates the risks of developing NAFLD according to the baseline WC quartiles stratified by sex. The average follow-up period for the participants who did not develop NAFLD was 5.4 years. In men, 4083 participants developed NAFLD during 91 664.2 person-years of follow-up (incidence density, 44.5 per 1000 person-years). In women, 2166 participants developed NAFLD during 105 799.7 person-years of follow-up (incidence density, 20.4 per 1000 person-years). We first analyzed the relationships between baseline WC and the incidence of NAFLD after only adjusting for age, and then we adjusted for age, smoking status, alcohol intake, regular exercise, educational level, and BMI. In both the age- and multivariate-adjusted models, higher baseline WC quartile predicted the incidence of NAFLD in a graded and dose-response manner (p for trend < 0.001). Compared with the lowest quartile (i.e., the reference group), the highest quartile was at a significantly higher risk of developing NAFLD (aHR = 1.81 and 95% CI = 1.59–2.07 in men; aHR = 2.61 and 95% CI = 2.15–3.18 in women). The overall interaction between sex and WC quartile for developing NAFLD was significant (p for interaction < 0.001). The association between WC change and the incidence of NAFLD tended to be stronger in women than in men. Further adjustments for glucose, systolic blood pressure, triglycerides, HDL-C, hsCRP, and HOMA-IR levels did not materially alter these estimates in men and women.

**Table 3:** Association between baseline waist circumference and the development of non-alcoholic fatty liver disease.

WC (cm) quartile	Person-years	Incident cases	Incidence rate (per 1000 person-years)	Age-adjusted HR (95% CI)	Multivariate HR (95% CI)	
					Model 1^a^	Model 2^b^
Men						
1^st^ Q (≤ 76.0)	25,759.70	573	22.2	1	1	1
2^nd^ Q (76.1–80.3)	21,924.40	891	40.6	1.89 (1.70–2.10)	1.50 (1.34–1.67)	1.44 (1.29–1.62)
3^rd^ Q (80.4–84.8)	22,192.70	1139	51.3	2.45 (2.21–2.70)	1.68 (1.50–1.88)	1.57 (1.39–1.76)
4^th^ Q (≥ 84.9)	21,787.30	1480	67.9	3.33 (3.02–3.67)	1.81 (1.59–2.07)	1.61 (1.41–1.85)
P for trend				<0.001	<0.001	<0.001
Women						
1^st^ Q (≤ 66.5)	28,841.90	178	6.2	1	1	1
2^nd^ Q (66.6–70.6)	27,676.80	356	12.9	2.05 (1.71–2.46)	1.48 (1.23–1.78)	1.42 (1.18–1.72)
3^rd^ Q (70.7–75.0)	25,820.80	618	23.9	3.85 (3.26–4.56)	2.18 (1.82–2.60)	2.01 (1.68–2.41)
4^th^ Q (≥ 75.1)	23,459.90	1014	43.2	7.24 (6.15–8.52)	2.61 (2.15–3.18)	2.31 (1.89–2.83)
P for trend				<0.001	<0.001	<0.001

Abbreviations: WC, waist circumference; HR, hazard ratio; CI, confidence intervals; HDL-C, high-density lipoprotein-cholesterol; hsCRP, high-sensitivity C-reactive protein; HOMA-IR, homeostasis model assessment of insulin resistance.

The overall interaction between sex and waist circumference quartile for non-alcoholic fatty liver disease development was <0.001.

^a^Model 1: adjusted for age, smoking status, alcohol intake, regular exercise, educational level, and body mass index.

^b^Model 2: adjusted for variables in model 1 plus glucose, systolic blood pressure, triglycerides, HDL-C, hsCRP, and HOMA-IR.

Table 4 shows the association between the incidence of NAFLD and WC change between visit 1 and visit 2. The timing of WC change assessment between visit 1 and visit 2 differed among study participants, and the average period between visit 1 and visit 2 was 1.9 years. In both the age- and multivariate-adjusted models, which included baseline WC and the time period between visits (in years), the risk for NAFLD increased as the WC change quartile increased (p for trend < 0.001). For men, the lowest quartile (WC loss ≤ -2.0 cm) demonstrated significantly decreased risk for NAFLD (aHR = 0.79; 95% CI = 0.73–0.87) while the highest quartile (WC gain) demonstrated significantly elevated risk for NAFLD (aHR = 1.30; 95% CI = 1.19–1.42) in comparison with the reference group (no change in WC). For women, the lowest quartile (WC loss) demonstrated significantly decreased risk for NAFLD (aHR = 0.72; 95% CI = 0.63–0.81) while the highest quartile (WC gain) demonstrated significantly elevated risk for NAFLD (aHR = 1.48; 95% CI = 1.31–1.67) in comparison with the reference group. The association between WC change and the incidence of NAFLD did not differ significantly according to sex (p for interaction = 0.067).

**Table 4:** Association between the development of non-alcoholic fatty liver disease and waist circumference change during the 2-year period between visit 1 and visit 2.

Quartile for WC change (cm) during the 2 years between visit 1 and visit 2	Person-years	Incident cases	Incidence rate (per 1000 person-years)	Age-adjusted HR (95% CI)	Multivariate HR (95% CI)	
					Model 1^a^	Model 2^b^
Men						
1^st^ Q (≤ -2.0)	17,243.00	1091	63.2	1	0.88 (0.80–0.96)	0.79 (0.73–0.87)
2^nd^ Q (-1.9–0.5)	13,158.40	914	69.4	1.08 (0.99–1.18)	1	1
3^rd^ Q (0.6–3.2)	14,302.10	960	67.1	1.04 (0.96–1.14)	1.00 (0.91–1.09)	1.03 (0.94–1.18)
4^th^ Q (≥ 3.3)	14,764.30	1118	75.7	1.20 (1.11–1.31)	1.16 (1.06–1.22)	1.30 (1.19–1.42)
P for trend				<0.001	<0.001	<0.001
Women						
1^st^ Q (≤ -1.5)	18,399.30	532	28.9	1	0.82 (0.73–0.93)	0.72 (0.63–0.81)
2^nd^ Q (-1.4–1.3)	17,108.70	499	29.1	1.01 (0.89–1.14)	1	1
3^rd^ Q (1.4–4.3)	16695.4	556	33.3	1.16 (1.03–1.31)	1.15 (1.01–0.29)	1.21 (1.07–1.36)
4^th^ Q (≥ 4.4)	15,653.20	579	36.9	1.35 (1.20–1.52)	1.29 (1.15–1.46)	1.48 (1.31–1.67)
P for trend				<0.001	<0.001	<0.001

Abbreviations: WC, waist circumference; HR, hazard ratio; CI, confidence intervals.

The overall interaction between sex and waist circumference quartile for the development of non-alcoholic fatty liver disease was 0.082.

^a^Model 1: adjusted for period (years) from visit 1 to visit 2, age, smoking status, alcohol intake, regular exercise, educational level, and body mass index.

^b^Model 2: adjusted for variables in model 1 plus for baseline waist circumference.

## Discussion

In the present cohort study of relatively healthy Korean men and women, both baseline WC and WC change over a 2-year period were positively associated with the development of NAFLD in a dose-response manner. Compared with stable WC, WC gain were associated with an increased risk for NAFLD, whereas WC loss was associated with a decreased risk of NAFLD. This relationship between WC change and the risk of NAFLD persisted regardless of the baseline WC and potential confounders. Thus, WC gain appears to be an independent risk factor for NAFLD, even in a relatively lean population.

To the best of our knowledge, this is the first study to demonstrate that WC change was positively associated with the risk of NAFLD. Most published studies on WC were cross-sectional investigations. A recent meta-analysis of central obesity and NAFLD showed that patients with central obesity (higher WC level) had a higher risk of NAFLD than individuals with general obesity (higher BMI level) [25]. Only one cohort study showed WC as a risk factor for NAFLD [18]. However, this study was limited by small sample size (n = 211) and did not distinguish newly-developed NAFLD from NAFLD progression. Concordant with previous studies, our findings indicate a positive relationship between baseline WC and the future risk of NAFLD, even after adjusting for baseline cardiometabolic markers. Moreover, WC increases over time also contribute to the incidence of NAFLD in both men and women after adjusting for baseline WC, supporting an independent role of waist change in the development of NAFLD.

Although the underlying mechanism has not been fully elucidated, waist gain may reflect more visceral fat accumulation. The International Study of Prediction of Intra-Abdominal Adiposity, a cross-sectional study, reported that WC is positively associated with VAT and liver fat in each BMI category [26]. A recent large cohort study has shown that larger areas of VAT are longitudinally associated with higher risk of incident NAFLD; in contrast, larger areas of SAT are longitudinally associated with regression of NAFLD [27]. VAT is metabolically active, and lipolysis in VAT may directly increase free fatty acid flux into the portal vein and liver [28]. Increased visceral adiposity may result in the accumulation of triglycerides in hepatocytes, which is the main pathogenic trigger for the development of NAFLD [29,30]. Increased visceral fat-induced cytokines, inflammation, and oxidative damage may also be associated with the development of NAFLD [9,31]. A recent study reported that visceral fat is independently associated with both necroinflammation and increased fibrosis on biopsy in NAFLD patients, and suggested that visceral fat deposition is a primary factor involved in the development of NAFLD [9]. In addition, some cytokines from VAT are involved in the transformation of hepatic cells to myofibroblastic phenotypes [32].

In our study, the association between WC change and the incidence of NAFLD was stronger in women, even though the absolute incidence of NAFLD was higher in men. Previous population-based studies reported a significantly higher prevalence of NAFLD in men than in women [33], possibly related to more favorable lifestyle factors and the protective effect of sex hormones in women [34,35]. The reasons for a stronger association of WC change with incident NAFLD in women are unclear, but glucose metabolism and central body fat distribution may be a more important contributor to fatty liver in women than in men [36]. Our study was conducted in asymptomatic relatively young and lean women. The mean (standard deviation) of age and BMI were 38.6 (7.0) years and 21.3 (2.3) kg/m^2^. Due to the small number of postmenopausal women in our study, the association between WC change and the incidence of NAFLD in women in our study reflects primarily findings in premenopausal women, and we were not able to perform stratified analysis by menopausal status. Further research is needed to understand this sex-related difference.

We observed that participants with WC loss demonstrated a decreased risk of NAFLD, despite higher baseline cardiometabolic parameters, including BMI, WC, and HOMA-IR, in comparison with the participants in the other quartiles. This suggests that even though the risk of NAFLD may be high at a certain point, WC loss may reduce risk. As a standard practice, individuals who present with larger BMI or WC or worse metabolic profiles are more likely to be encouraged to implement dietary changes, exercise interventions, or smoking cessation, compared to those who have healthy profiles. However, because no information patients’ intention to WC reduction was recorded, we could not differentiate between intentional and unintentional WC loss. The present study demonstrates that enlargement of WC predicts the development of NAFLD and suggests that metabolic syndrome may be prevented by decreasing WC [37].

Our current study had some limitations. First, histologic confirmations were not used to diagnose NAFLD in our study series. In addition, ultrasonography findings were not scored semi-quantitatively according to severity of NAFLD [10]. However, many population-based epidemiologic studies diagnose a fatty liver using ultrasonography because noninvasive imaging tests, including ultrasound, are recognized as reliable tools for this purpose [10,38]. Second, in practical health screening, it was hard to measure the abdominal adiposity by more accurate and costly methods, such as computed tomography, dual-energy X-ray absorptiometry, and magnetic resonance imaging. Finally, our study was performed in asymptomatic relatively young and lean Korean adults and our findings cannot be generalized to other populations. Nevertheless, in this substantial sample of population, our study is the first prospective cohort study to demonstrate the association between baseline WC, WC changes and the development of NAFLD.

In conclusion, WC gain as well as baseline WC appears to be major contributing factors to the development of NAFLD in adults. Our current findings also suggest that avoiding WC gain can prevent the risk of NAFLD regardless of baseline WC. Further research is needed to elucidate and validate the mechanisms underlying these relationships and design intervention programs for WC reduction.
